# Predicting CKD progression using time-series clustering and light gradient boosting machines

**DOI:** 10.1038/s41598-024-52251-9

**Published:** 2024-01-19

**Authors:** Hirotaka Saito, Hiroki Yoshimura, Kenichi Tanaka, Hiroshi Kimura, Kimio Watanabe, Masaharu Tsubokura, Hiroki Ejiri, Tianchen Zhao, Akihiko Ozaki, Sakumi Kazama, Michio Shimabukuro, Koichi Asahi, Tsuyoshi Watanabe, Junichiro J. Kazama

**Affiliations:** 1 Department of Nephrology and Hypertension, Fukushima Medical University, 1 Hikariga-Oka, Fukushima City, Fukushima 960-1295 Japan; 2 Department of Radiation Health Management, Fukushima Medical University, Fukushima, Japan; 3 Division of Advanced Community Based Care for Lifestyle Related Diseases, Fukushima Medical University, Fukushima, Japan; 4 Department of Thyroid and Endocrinology, Fukushima Medical University, Fukushima, Japan; 5 Department of Diabetes, Endocrinology, and Metabolism, Fukushima Medical University, Fukushima, Japan; 6 Division of Nephrology and Hypertension, Iwate Medical University, Yahaba, Japan

**Keywords:** Diseases, Nephrology, Risk factors

## Abstract

Predicting the transition of kidney function in chronic kidney disease is difficult as specific symptoms are lacking and often overlooked, and progress occurs due to complicating factors. In this study, we applied time-series cluster analysis and a light gradient boosting machine to predict the trajectories of kidney function in non-dialysis dependent chronic kidney disease patients with baseline estimated glomerular filtration rate (GFR) ≥ 45 mL/min/1.73 m^2^. Based on 5-year changes in estimated GFR, participants were stratified into groups with similar trajectories by cluster analysis. Next, we applied the light gradient boosting machine algorithm and Shapley addictive explanation to develop a prediction model for clusters and identify important parameters for prediction. Data from 780 participants were available for analysis. Participants were classified into five classes (Class 1: n = 78, mean [± standard deviation] estimated GFR 100 ± 19.3 mL/min/1.73 m^2^; Class 2: n = 176, 76.0 ± 9.3 mL/min/1.73 m^2^; Class 3: n = 191, 59.8 ± 5.9 mL/min/1.73 m^2^; Class 4: n = 261, 52.7 ± 4.6 mL/min/1.73 m^2^; and Class 5: n = 74, 53.5 ± 12.0 mL/min/1.73 m^2^). Declines in estimated GFR were 8.9% in Class 1, 12.2% in Class 2, 4.9% in Class 3, 12.0% in Class 4, and 45.1% in Class 5 during the 5-year period. The accuracy of prediction was 0.675, and the top three most important Shapley addictive explanation values were 1.61 for baseline estimated GFR, 0.12 for hemoglobin, and 0.11 for body mass index. The estimated GFR transition of patients with preserved chronic kidney disease mostly depended on baseline estimated GFR, and the borderline for estimated GFR trajectory was nearly 50 mL/min/1.73 m^2^.

## Introduction

Chronic kidney disease (CKD) is a global public health problem defined as kidney damage or glomerular filtration rate (GFR) < 60 mL/min/1.73 m^2^ for 3 months according to the Kidney Disease Improving Global Outcomes CKD guidelines^1^. CKD is caused by various diseases, including hypertension, diabetes, obesity, or glomerulonephritis, and is associated with adverse outcomes including cardiovascular events, end-stage kidney disease (ESKD), and death. Preventing the progression of CKD is thus crucial for public health and reducing medical costs. However, predicting the transition of kidney function in CKD patients is challenging, as most patients do not exhibit symptoms when kidney function is preserved, and the speed of progression of kidney dysfunction is affected by complicating factors.

Recently, the application of machine learning methods in medical research has become increasingly widespread. Machine learning mainly performs regression, classification and cluster analyses. Regression is to predict continuous data, classification is to predict categorical data, and clustering is to separate data into subgroups based on specific characteristics. Regression and classification can be considered as an application or example of supervised learning, where the algorithm is trained on labeled dataset, which means that the input data used for training is paired with corresponding output or target values. On the other hand, clustering is categorized as unsupervised learning which deals with data that lacks explicit labels or targets to discover patterns, relationships, or structures within the data without the guidance of predefined labels or categories.

To date, several studies have reported machine learning models in relation to CKD. Some studies have predicted risk factors for ESKD in patients with CKD^2–6^, rapid declines in estimated GFR (eGFR) from laboratory data^7–9^, or progression of CKD from imaging features derived from kidney ultrasounds^10^, and other studies have predicted the development of CKD in the general population or diabetes patients^11–13^. However, to the best of our knowledge, no study has conducted both cluster analysis and supervised machine learning to predict eGFR transition. Here we combined time-series cluster analysis and supervised learning, applying a light gradient boosting machine (GBM) algorithm to predict the transition of eGFR and identify important features for CKD progression in CKD patients with relatively preserved kidney function using baseline characteristic data from the Fukushima CKD Cohort Study.

## Materials and methods

### Study population

The Fukushima CKD Cohort Study^14–17^, as a sub-cohort of the Fukushima Cohort Study, is a prospective survey investigating the characteristics and clinical outcomes (such as cardiovascular events, ESKD, and death) of non-dialysis-dependent patients with CKD at Fukushima Medical University Hospital, Japan. The study was registered in the University Hospital Medical Information Network Clinical Trials Registry (UMIN-CTR UMIN000040848). Participants were recruited between June 2012 and July 2014, with a total of 2724 participants registered for the Fukushima Cohort Study. Inclusion criteria were as follows: (1) Japanese patients living in Japan; (2) age ≥ 18 years; and (3) CKD according to the definition of eGFR < 60 mL/min/1.73 m^2^ or positive dipstick results for proteinuria (≥ 1 +), with stable renal function for ≥ 3 months before entry into the study. The calculation of eGFR was performed using the estimation equation for Japanese patients with CKD^18^. Exclusion criteria were as follows: (1) dialysis treatment within the last 3 months; (2) active malignancy; (3) infectious disease; (4) pregnancy; or (5) history of organ transplantation. We also excluded patients for whom data on serum creatinine were missing. Among patients in the Fukushima CKD Cohort Study, subjects with baseline eGFR ≥ 45 mL/min/1.73 m^2^ and measurement of eGFR at least two times during follow-up were extracted for analysis. The protocol was approved by the Ethics Committee of Fukushima Medical University (approval no. 2001), and the study was conducted in accordance with the Declaration of Helsinki. All patients provided written, informed consent.

### Data collection

Information regarding demographics, comorbidities, and medications at baseline was obtained from the medical records or blood examination results at registration. Body mass index was calculated as weight in kilograms divided by height in meters squared, and obesity was defined as body mass index ≥ 25 kg/m^2^. Blood pressure was measured by trained staff using a standard sphygmomanometer or an automated device with the patient in a sitting position. Pulse pressure was calculated as systolic blood pressure minus diastolic blood pressure. Hypertension was defined as: (1) systolic blood pressure ≥ 140 mmHg; or (2) diastolic blood pressure ≥ 90 mmHg; or (3) the use of antihypertensive medications. Diabetes mellitus was identified as: (1) fasting plasma glucose concentration ≥ 126 mg/dL; (2) hemoglobin A1c (National Glycohemoglobin Standardization Program) ≥ 6.5%; or (3) use of insulin or oral antihyperglycemic drugs. Dyslipidemia was defined as: (1) triglycerides ≥ 150 mg/dL; (2) low-density lipoprotein (LDL)-cholesterol concentration ≥ 140 mg/dL; (3) high-density lipoprotein (HDL)-cholesterol concentration < 40 mg/dL; or (4) use of antihyperlipidemic medications. Hyperuricemia was defined as: (1) serum uric acid ≥ 8.0 mg/dL; or (2) use of uric acid-lowering drugs. During up to 5 years of follow-up, eGFR was measured annually until censoring, development of ESKD, or death.

### Statistical analyses

Categorical variables were counted for each category, including deficits, and continuous variables are presented as the mean and standard deviation. Values were compared using one-way analysis of variance or the χ^2^ test, as appropriate.

### Time-series cluster analysis

To classify participants into subgroups according to changes in eGFR during follow-up, we applied hierarchical cluster analysis using the method of Ward, measuring Euclidean distance. Analysis was conducted based on a 5-year change in eGFR. In cases where data on eGFR were lacking due to censorship, development of ESKD, death, failure to attend medical appointments, or a lack of measurements from a particular visit, data were imputed by linear interpolation.

### LightGBM

Based on the results of cluster analysis, LightGBM, a supervised machine learning algorithm, was applied to develop a prediction model to identify informative variables for CKD progression. In LightGBM, missing values are ignored during a split and allocated to whichever side reduces the loss the most. Multinomial outcomes for the prediction model were defined as Classes 1–5, as generated by cluster analysis. The dataset was randomly allocated into separate training and test data: 70% of data were used for training the model then the remaining 30% were used to test the predictive performance of the model. The ratio of each class made by cluster analysis was kept in each separate data as an overall dataset. The prediction model was trained using baseline characteristic data for covariates. We tested three patterns of covariates to select the best model for detailed examination. Model 1 dealt with values seemed to be essential for the prediction, Model 2 dealt with all value seemed to be related with the prediction (full set), and Model 3 dealt with values seemed to be affecting the prediction according to the result of prediction by Model 1 and 2. Covariates were selected as follows: Model 1 included age, sex, body mass index, smoking history, systolic blood pressure, diastolic blood pressure, history of cardiovascular disease, diabetes, eGFR, serum albumin, hemoglobin, qualitative test for proteinuria, and use of angiotensin-converting enzyme (ACE) inhibitor or angiotensin II receptor blocker (ARB). Model 2 included age, sex, body mass index, obesity, smoking history, systolic blood pressure, diastolic blood pressure, pulse pressure, heart rate, history of cardiovascular disease, hypertension, diabetes, dyslipidemia, hyperuricemia, eGFR, corrected calcium, phosphorus, LDL-cholesterol, serum albumin, white blood cell count, hemoglobin, platelet count, mean corpuscular volume (MCV), mean corpuscular hemoglobin (MCH), MCH concentration (MCHC), red cell distribution width (RDW), qualitative test for proteinuria, and use of medicine (ACE inhibitor or ARB, calcium channel blocker, loop diuretic, thiazide diuretic, spironolactone, statin, xanthine oxidase inhibitor, aspirin, warfarin, or proton pump inhibitor). Model 3 included age, sex, body mass index, smoking history, systolic blood pressure, diastolic blood pressure, history of cardiovascular disease, diabetes, eGFR, LDL-cholesterol, serum albumin, hemoglobin, platelet count, MCHC, RDW, qualitative test for proteinuria, use of an ACE inhibitor or ARB, and use of a xanthine oxidase inhibitor. Multi-error was used to assess the accuracy of classification. To validate correlations between classification and covariates, regression analysis was also performed by LightGBM, in which each class was replaced as a discrete variable. Mean absolute error was used to assess accuracy. Shapley additive explanation (SHAP, version 0.41.0) was used to interpret the results of prediction by LightGBM (version 3.3.2). Training data set was used to calculate SHAP values. In LightGBM, we set "learning_rate" to 0.01, "boosting_type" to "gbdt", "objective" to "multiclass", "metric" to "multi_error", and "num_boost_round" to "50,000". All other settings were left as default. All analyses were conducted using Python version 3.9.12 (Python Software Foundation Inc.).

## Results

**Figure 1:**
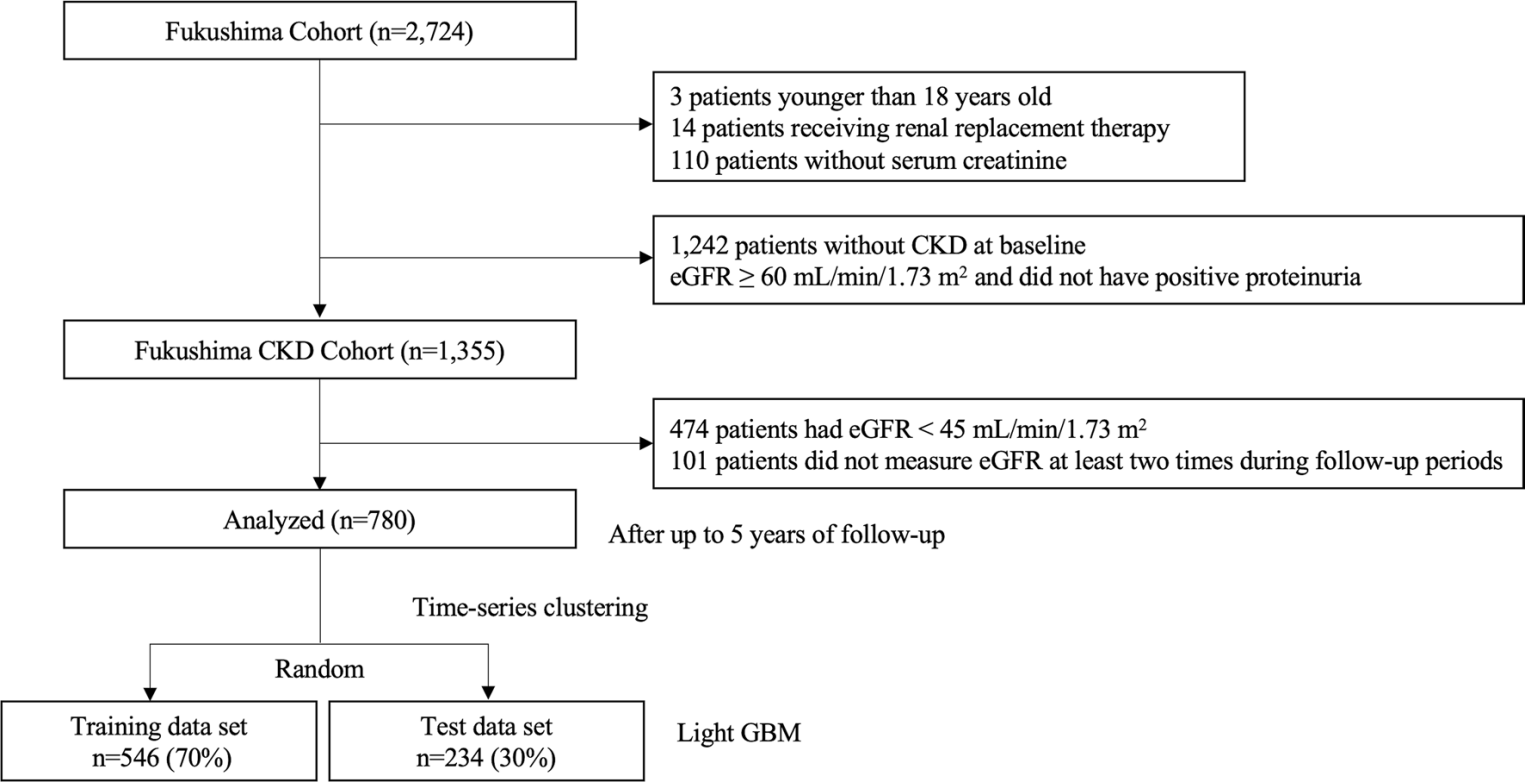
Flow of participants in the present study. *CKD* chronic kidney disease, *eGFR* estimated glomerular filtration rate, *GBM* gradient boosting machine.

A total of 780 participants were extracted and analyzed from the 1,355 participants in the Fukushima CKD Cohort dataset (Fig. 1). Measurements of eGFR were obtained for a maximum of 5 years after registration. Baseline characteristics are shown in Table 1. Mean age was 61.7 years, 57.1% were male, mean eGFR [± SD] was 64.9 ± 17.8 mL/min/1.73 m^2^.

**Table 1:** Characteristics of the study patients (all and Classes 1–5 according to time-series cluster analysis).

Variables (number of missing data)	All patients	Class 1	Class 2	Class 3	Class 4	Class 5	P value for trend	P value class 4 vs. 5
n	780	78	176	191	261	74		
Age (years) (0)	61.7 ± 14.6	47.3 ± 16.1	55.8 ± 14.1	64.5 ± 12.3	66.1 ± 10.3	66.6 ± 13.0	< 0.001	0.72
Male sex (%) (0)	57.1	60.3	56.8	58.1	54.0	60.8	0.77	0.37
Body mass index (kg/m^2^) (28)	25.3 ± 5.2	26.7 ± 8.2	25.7 ± 4.9	25.2 ± 4.7	24.8 ± 4.4	25.5 ± 5.6	0.03	0.23
Smoking history (%) (48)	50.6	60.3	53.4	48.7	44.1	62.2	0.03	0.008
Cardiovascular disease (%) (0)	10.2	6.4	8.0	7.9	11.9	18.9	0.04	0.17
Diabetes mellitus (%) (0)	48.0	57.7	44.9	44.5	49.0	59.5	0.08	0.15
Hypertension (%) (0)	81.7	70.5	78.4	81.2	85.8	87.8	0.01	0.80
Dyslipidemia (%) (0)	67.6	53.8	64.8	61.6	70.5	77.0	0.09	0.41
Hyperuricemia (%) (0)	33.1	20.5	25.0	28.8	39.5	54.1	< 0.001	0.05
Systolic blood pressure (mmHg) (1)	131.1 ± 18.5	128.2 ± 19.0	134.0 ± 16.7	132.2 ± 17.9	130.5 ± 18.7	133.9 ± 20.9	0.1	0.18
Diastolic blood pressure (mmHg) (1)	76.1 ± 12.1	76.7 ± 11.1	79.1 ± 11.1	76.2 ± 11.1	75.3 ± 11.9	75.6 ± 15.3	0.02	0.83
Pulse pressure (mmHg) (1)	55.3 ± 15.4	54.5 ± 15.0	54.9 ± 14.8	56.1 ± 15.6	55.3 ± 15.7	58.3 ± 14.9	0.1	0.14
Heart rate (/min) (2)	77.1 ± 13.4	80.9 ± 14.2	77.4 ± 13.3	74.4 ± 12.2	77.4 ± 13.9	78.0 ± 13.3	0.06	0.73
eGFR (mL/min/1.73 m^2^) (0)	64.9 ± 17.8	100.4 ± 19.3	76.0 ± 9.3	59.8 ± 5.9	52.7 ± 4.6	53.5 ± 12.0	< 0.001	0.43
Proteinuria (%) (19)							< 0.001	0.004
(−)	32.9	0.0	2.3	37.7	58.2	40.5		
(1 +)	33.5	62.8	51.1	33.0	17.6	17.6		
(2 +)	19.8	26.9	26.7	17.3	14.6	20.3		
(3 +)	9.1	9.0	17.0	8.4	5.0	8.1		
(4 +)	2.6	1.3	2.8	0.5	2.3	10.8		
Corrected calcium (mg/dL) (287)	9.5 ± 0.82	9.8 ± 1.2	9.4 ± 0.5	9.5 ± 0.9	9.5 ± 0.9	9.6 ± 0.8	0.005	0.53
Phosphorus (mg/dL) (348)	3.2 ± 0.58	3.3 ± 0.6	3.2 ± 0.5	3.2 ± 0.6	3.2 ± 0.6	3.3 ± 0.6	0.14	0.94
HDL-cholesterol (mg/dL) (42)	53.8 ± 14.7	49.8 ± 12.5	56.5 ± 14.2	54.0 ± 14.9	54.2 ± 15.4	48.9 ± 13.3	< 0.001	0.009
LDL-cholesterol (mg/dL) (56)	107.9 ± 29.7	110.0 ± 30.7	109.9 ± 28.8	109.2 ± 29.9	106.7 ± 27.5	102.1 ± 36.9	0.99	0.25
Serum albumin (g/dL) (109)	4.0 ± 0.5	4.0 ± 0.5	4.0 ± 0.4	4.0 ± 0.4	4.0 ± 0.4	3.8 ± 0.6	< 0.001	< 0.001
Hemoglobin (g/dL) (24)	13.4 ± 1.7	13.6 ± 1.9	13.8 ± 1.5	13.6 ± 1.8	13.2 ± 1.5	12.5 ± 1.9	< 0.001	0.001
White blood cell count (× 10^3^/μL) (22)	6.3 ± 2.1	6.7 ± 2.6	6.4 ± 2.0	6.1 ± 2.1	6.2 ± 2.0	6.4 ± 2.2	0.31	0.44
Platelet count (× 10^4^/μL) (25)	21.3 ± 11.3	26.7 ± 30.8	21.8 ± 5.6	20.6 ± 6.2	20.0 ± 5.2	20.4 ± 6.3	0.05	0.58
MCV (fL) (21)	93.1 ± 5.4	90.8 ± 8.0	92.7 ± 4.6	93.1 ± 5.5	93.9 ± 4.6	93.5 ± 5.6	0.02	0.51
MCHC (%) (21)	33.7 ± 0.8	33.5 ± 0.9	33.7 ± 0.7	33.7 ± 0.9	33.6 ± 0.7	33.6 ± 1.0	0.13	0.97
RDW (%) (17)	13.8 ± 1.5	14.1 ± 1.7	13.5 ± 1.2	13.7 ± 1.5	13.7 ± 1.4	14.5 ± 1.8	0.001	< 0.001
ACE inhibitor or ARB (%) (0)	63.7	52.6	64.2	62.8	68.2	75.7	0.03	0.27
Calcium channel blocker (%) (0)	45.6	32.1	43.8	44.0	47.9	60.8	0.008	0.07
Loop diuretic (%) (0)	6.5	2.6	5.7	4.7	6.5	17.6	0.001	0.007
Thiazide diuretic (%) (0)	10.9	6.4	9.1	10.5	13.0	13.5	0.41	1
Spironolactone (%) (0)	6.5	1.3	3.4	5.8	7.7	17.6	< 0.001	0.02
Statin (%) (0)	47.1	30.8	43.8	50.3	51.3	48.6	0.02	0.78
Xanthine oxidase inhibitor (%) (0)	18.2	10.3	12.5	13.6	23.0	35.1	< 0.001	0.05
Aspirin (%) (0)	13.3	10.3	13.6	10.5	13.4	23.0	0.09	0.07
Warfarin (%) (0)	6.4	3.8	5.1	6.3	6.9	10.8	0.43	0.39
Proton pump inhibitor (%) (0)	20.4	20.5	21.6	18.3	20.3	23.0	0.92	0.74

*eGFR* estimated glomerular filtration rate, *HDL* high-density lipoprotein, *LDL* low-density lipoprotein, *MCV* mean corpuscular volume, *MCHC* mean corpuscular hemoglobin concentration, *RDW* red cell distribution width, *ACE* angiotensin-converting enzyme, *ARB* angiotensin II receptor blocker.

Time-series cluster analysis divided the 780 participants into 5 classes based on annual changes in eGFR over 5 years. The number of participants in each class was 78 (60.2% male) in Class 1, 176 (56.8% male) in Class 2, 191 (58.1% male) in Class 3, 261 (54.0% male) in Class 4, and 74 (60.8% male) in Class 5. Baseline characteristics of each class are shown in Table 1. Participants were older in the higher numbered groups, serum albumin and hemoglobin were decreased in Class 5, and eGFR was higher in the lower numbered groups.

**Figure 2:**
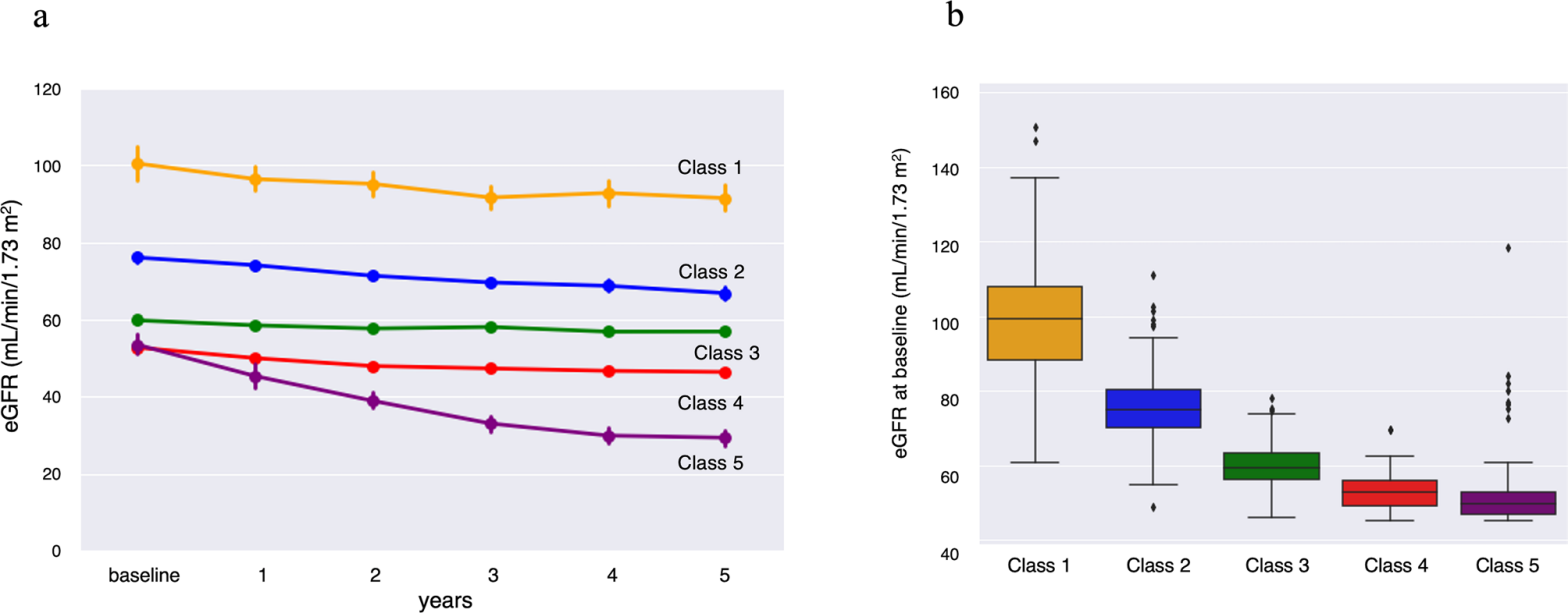
Trajectories of 5-year eGFR in each subgroup according to cluster analysis (**a**). Box plot of baseline eGFR in each subgroup according to cluster analysis (**b**). *eGFR* estimated glomerular filtration rate.

**Figure 3:**
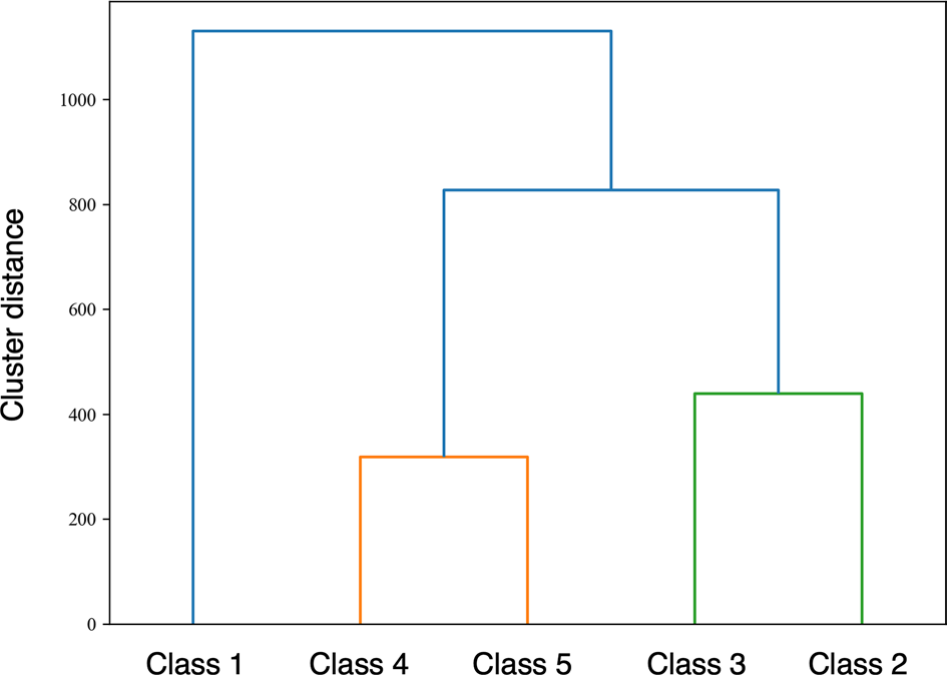
Dendrogram of cluster classification across 5 classes. Vertical axis indicates distance between the clusters.

Figure 2a shows the annual change in mean eGFR by class, and Fig. 2b shows box plots of baseline eGFR. Decline in eGFR over 5 years were 8.9% in Class 1, 12.2% in Class 2, 4.9% in Class 3, 12.0% in Class 4, and 45.1% in Class 5. The dendrogram of cluster classification across 5 classes is shown in Fig. 3. Baseline eGFR was almost the same in Classes 4 and 5, but the reduction in eGFR was markedly larger in Class 5.

**Figure 4:**
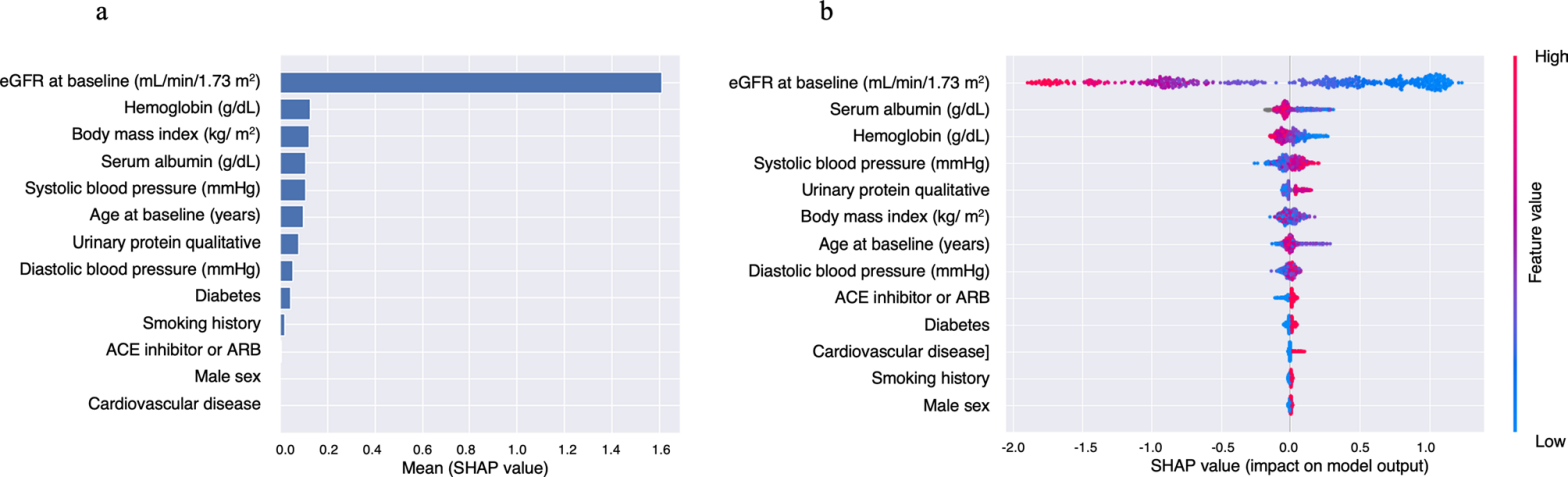
Importance matrix plot of the LightGBM model, representing the importance of each covariate (Model 1) for predicting classification of 5-year eGFR trajectory (**a**). SHAP summary plot of clinical features of the LightGBM model (Model 1). One dot represents a patient’s colored feature value, where red has a higher value and blue has a lower value. SHAP values that exceed zero indicate an increased risk for higher class (**b**). Model 1 adjusted for age, sex, body mass index, smoking history, systolic blood pressure, diastolic blood pressure, histories of cardiovascular disease and diabetes, eGFR, serum albumin, hemoglobin, qualitative test for proteinuria, and use of an ACE inhibitor or ARB. *eGFR* estimated glomerular filtration rate, *GBM* gradient boosting machine, *SHAP* Shapley additive explanation, *ACE* angiotensin-converting enzyme, *ARB* angiotensin II receptor blocker.

The accuracy of prediction of cluster classification across 5 classes by LightGBM were estimated for 3 models. Covariates included in these 3 models are shown in Table 2. The accuracies of prediction were 0.675, 0.645, and 0.641 for Model 1, 2, and 3, respectively, and the highest accuracy was observed in Model 1. Table 3 shows the multi-class confusion matrix for Model 1. The multi-class confusion matrices for Model 2 and 3 were shown in Supplemental Tables 1 and 2, respectively. Figure 4a shows the importance matrix plot ranking the covariates (Model 1) that contributed to the prediction of cluster classification by LightGBM. The top 5 SHAP values were 1.61 for eGFR, 0.13 for hemoglobin, 0.12 for body mass index, 0.11 for albumin, and 0.11 for systolic blood pressure. The SHAP summary plot shows the correlation between classification and covariates (Model 1), in which one dot represents a patient’s colored feature value, where red has a higher value and blue has a lower value. SHAP values that exceed zero indicate an increased risk for higher class (Fig. 4b). Each of eGFR, serum albumin, and hemoglobin were negatively related to classification, while systolic blood pressure and urinary protein qualitative were positively related. As the trajectory of eGFR was rather strongly influenced by baseline eGFR, these analyses were also performed in a model without baseline eGFR. In the model removing baseline eGFR from Model 1, the top 5 SHAP values were 1.14 for urinary protein qualitative, 0.84 for age, 0.47 for hemoglobin, 0.40 for systolic blood pressure, and 0.39 for body mass index (Supplemental Fig. 1).

**Table 2:** Covariates included in 3 models.

Variables	Model 1	Model 2	Model 3
Age	✓	✓	✓
Sex	✓	✓	✓
Body mass index	✓	✓	✓
Obesity		✓	
Smoking history	✓	✓	✓
Systolic blood pressure	✓	✓	✓
Diastolic blood pressure	✓	✓	✓
Pulse pressure		✓	
Heart rate		✓	
Cardiovascular disease	✓	✓	✓
Hypertension		✓	
Diabetes	✓	✓	✓
Dyslipidemia		✓	
Hyperuricemia		✓	
eGFR	✓	✓	✓
Corrected calcium		✓	
Phosphorus		✓	
LDL-cholesterol		✓	✓
Serum albumin	✓	✓	✓
White blood cell count		✓	
Hemoglobin	✓	✓	✓
Platelet count		✓	✓
MCV		✓	
MCH		✓	
MCHC		✓	✓
RDW		✓	✓
Qualitative test for proteinuria	✓	✓	✓
ACE inhibitor or ARB	✓	✓	✓
Calcium channel blocker		✓	
Loop diuretic		✓	
Thiazide diuretic		✓	
Spironolactone		✓	
Statin		✓	
Xanthine oxidase inhibitor		✓	✓
Aspirin		✓	
Warfarin		✓	
Proton pump inhibitor		✓	

*eGFR* estimated glomerular filtration rate, *HDL* high-density lipoprotein, *LDL* low-density lipoprotein, *MCV* mean corpuscular volume, *MCHC* mean corpuscular hemoglobin concentration, *RDW* red cell distribution width, *ACE* angiotensin-converting enzyme, *ARB* angiotensin II receptor blocker.

**Table 3:** Multi-class confusion matrix for model 1.

Predicted classification	Actual classification					Total
	Class 1	Class 2	Class 3	Class 4	Class 5	
Class 1	16	2	0	0	0	18
Class 2	7	47	7	0	2	63
Class 3	1	3	28	11	0	43
Class 4	0	1	22	67	20	110
Class 5	0	0	0	0	0	0
Total	24	53	57	78	22	234

**Figure 5:**
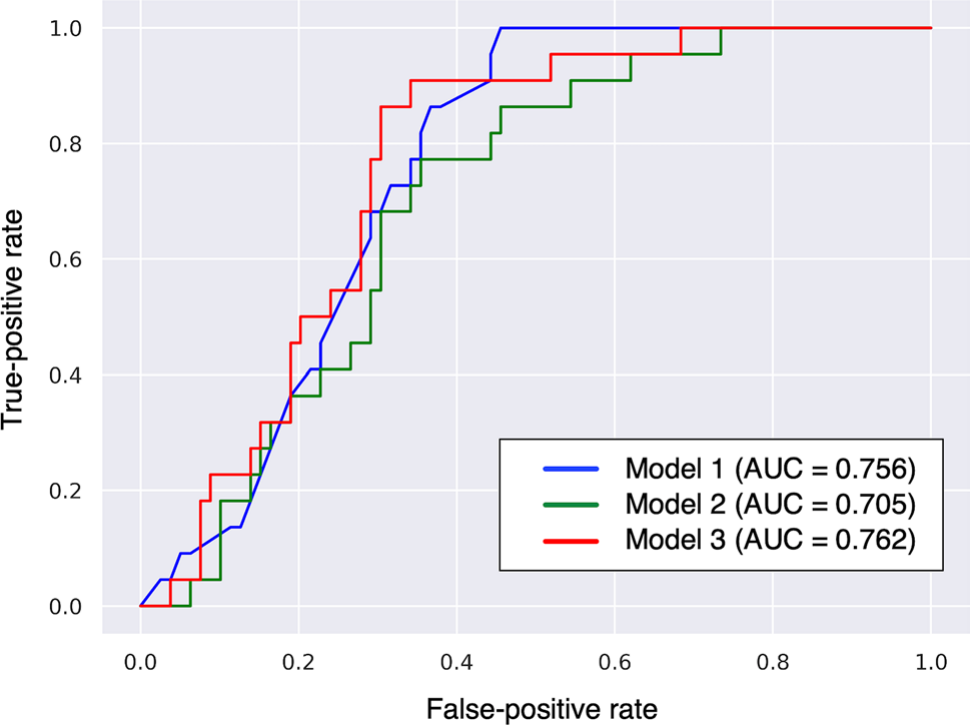
Comparison of AUC on prediction of whether a patient is allocated to Class 4 or 5 by three patterns of covariates. Model 1 adjusted for age, sex, body mass index, smoking history, systolic blood pressure, diastolic blood pressure, histories of cardiovascular disease and diabetes, eGFR, serum albumin, hemoglobin, qualitative test for proteinuria, and use of an ACE inhibitor or ARB; Model 2 adjusted for Model 1 plus pulse pressure, heart rate, hypertension, dyslipidemia, hyperuricemia, corrected calcium, phosphorus, LDL cholesterol, white blood cell, platelet, MCV, MCH, MCHC, RDW, use of medicine (calcium channel blocker, loop diuretic, thiazide diuretic, spironolactone, statin, xanthine oxidase inhibitor, aspirin, warfarin, or proton pump inhibitor); Model 3 adjusted for age, sex, body mass index, smoking history, systolic blood pressure, diastolic blood pressure, history of cardiovascular disease, diabetes, eGFR, LDL-cholesterol, serum albumin, hemoglobin, platelet count, MCHC, RDW, qualitative test for proteinuria, use of an ACE inhibitor or ARB, and use of a xanthine oxidase inhibitor. *AUC* area under the curve, *eGFR* estimated glomerular filtration rate, *ACE* angiotensin-converting enzyme, *ARB* angiotensin II receptor blocker, *LDL* low-density lipoprotein, *MCV* mean corpuscular volume, *MCH* mean corpuscular hemoglobin, *MCHC* mean corpuscular hemoglobin, *RDW* red cell distribution width.

**Figure 6:**
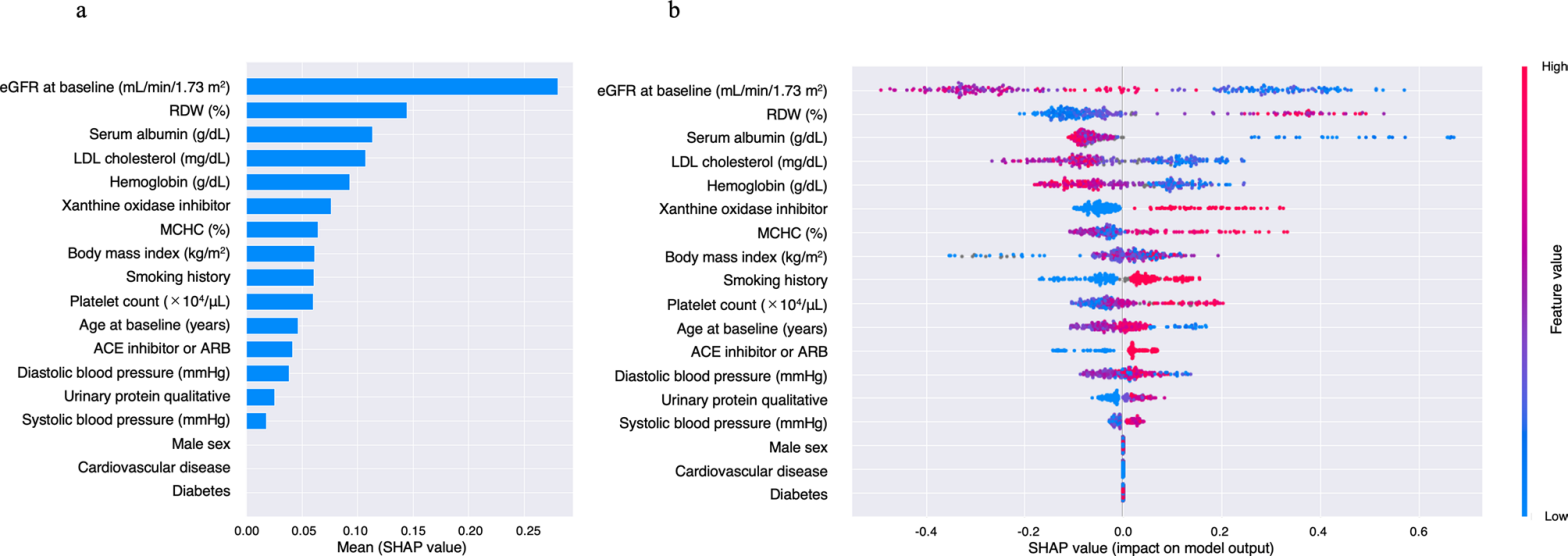
Importance matrix plot of the LightGBM model in Class 4 vs. Class 5 patients, representing the importance of each covariate (Model 3) for predicting classification of 5-year eGFR trajectory (**a**). SHAP summary plot of clinical features of the LightGBM model in Class 4 and 5 patients. One dot represents a patient’s colored feature value, where red has a higher value and blue has a lower value (**b**). Model 3 adjusted for age, sex, body mass index, smoking history, systolic blood pressure, diastolic blood pressure, history of cardiovascular disease, diabetes, eGFR, LDL-cholesterol, serum albumin, hemoglobin, platelet count, MCHC, RDW, qualitative test for proteinuria, use of an ACE inhibitor or ARB, and use of a xanthine oxidase inhibitor. *GBM* gradient boosting machine, *eGFR* estimated glomerular filtration rate, *LDL* low-density lipoprotein, *MCHC* mean corpuscular hemoglobin, *RDW* red cell distribution width, *ACE* angiotensin-converting enzyme, *ARB* angiotensin II receptor blocker.

**Figure 7:**
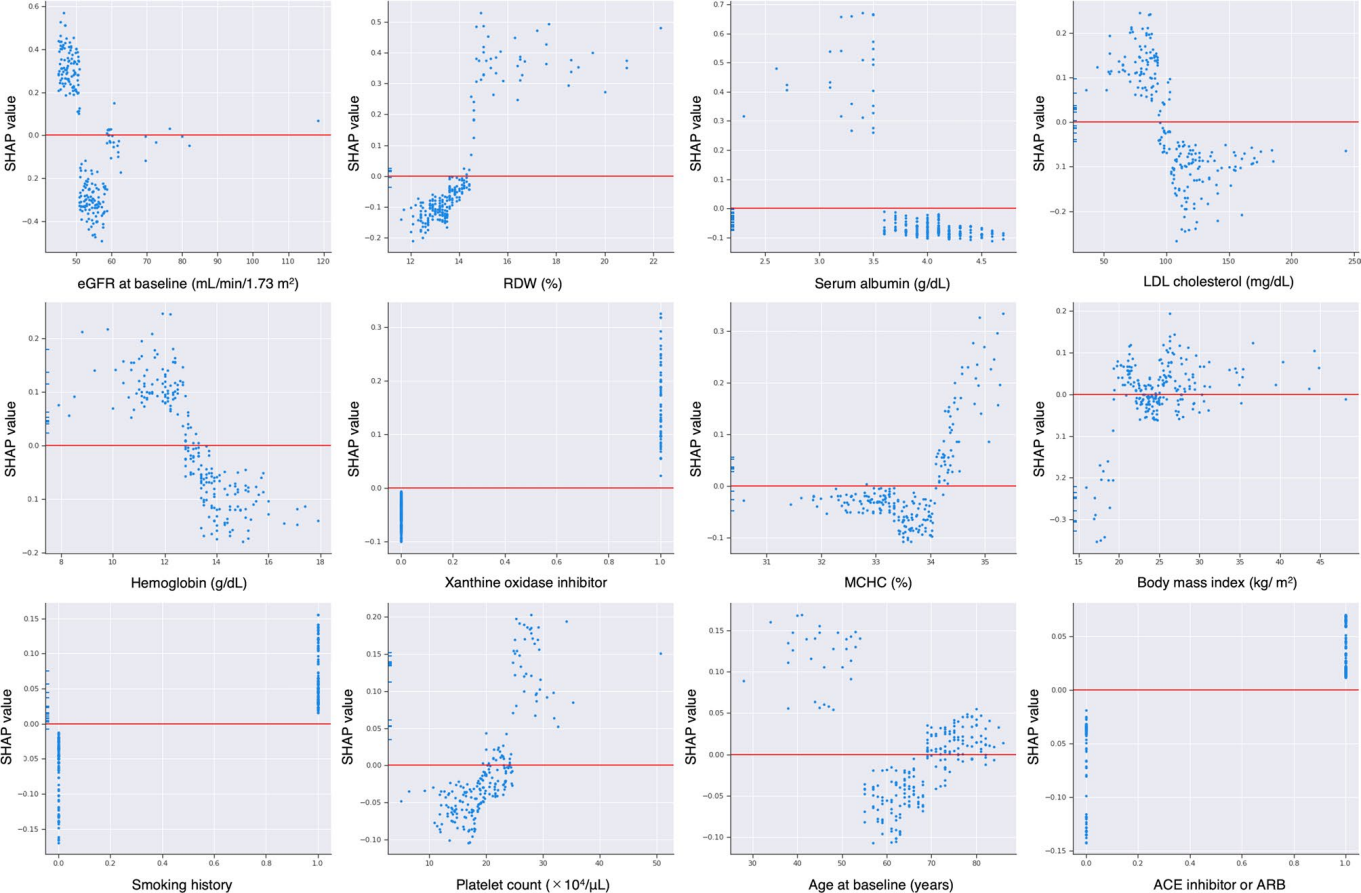
SHAP dependence plot of the LightGBM model, showing how a single variable affects prediction (Model 3). SHAP values that exceed zero indicate an increased risk for Class 5. Model 3 adjusted for age, sex, body mass index, smoking history, systolic blood pressure, diastolic blood pressure, history of cardiovascular disease, diabetes, eGFR, LDL-cholesterol, serum albumin, hemoglobin, platelet count, MCHC, RDW, qualitative test for proteinuria, use of an ACE inhibitor or ARB, and use of a xanthine oxidase inhibitor. *SHAP* Shapley additive explanation, *GBM* gradient boosting machine, *eGFR* estimated glomerular filtration rate, *RDW* red cell distribution width, *LDL* low-density lipoprotein, *MCHC* mean corpuscular hemoglobin concentration, *ACE* angiotensin-converting enzyme, *ARB* angiotensin II receptor blocker.

For predicting whether a patient would be allocated to Class 4 or 5, additional analysis was conducted on Class 4 and 5 patients excluding Class 1 to 3 patients. According to the AUC of ROC in Fig. 5, the highest accuracy of prediction was observed in covariate Model 3. The importance matrix plot of the LightGBM model (Model 3) indicated that RDW, serum albumin, LDL-cholesterol, and hemoglobin had higher importance values (SHAP values of 0.14 for RDW, 0.11 for serum albumin, 0.11 for LDL-cholesterol, and 0.09 for hemoglobin) after baseline eGFR (SHAP value 0.28) for predicting whether a patient would be allocated to Class 4 or 5 (Fig. 6a). The prediction of Class 5 correlated positively with RDW and negatively with eGFR, albumin, LDL-cholesterol, and hemoglobin (Fig. 6b). The SHAP dependence plot for the LightGBM model shows how each covariate (Model 3) influences the prediction of whether a patient belonged to Class 4 or 5, and SHAP values exceeding zero reflected an increased risk of Class 5 status (Fig. 7). Patient with an eGFR ≥ 45 mL/min/1.73 m^2^ but < 50 mL/min/1.73 m^2^, RDW ≥ 14.5%, lower albumin, lower LDL-cholesterol, lower hemoglobin, higher MCHC, history of smoking, use of a xanthine oxidase inhibitor and use of an ACE inhibitor or ARB showed an increased risk of Class 5 status (Fig. 7). In the model removing baseline eGFR from Model 3, the top 5 SHAP values were 0.26 for smoking history, 0.23 for hemoglobin, 0.21 for body mass index, 0.17 for serum albumin, and 0.13 for age (Supplemental Fig. 2).

## Discussion

In this study, we demonstrated that CKD patients with relatively preserved kidney function can be classified into five subgroups based on transitions of 5-year eGFR using time-cluster analysis. Furthermore, we found that the trajectory of eGFR was mostly dependent on baseline eGFR using a prediction model based on LightGBM. Clustering is an unsupervised data-mining technique used to group similar data into related or homogeneous groups and time-series modeling is used to establish the connection between observed data and future events. In the field of CKD, Bi et al. estimated dry weight of hemodialysis patients using a time series-based regression method^19^, and Ye et al. investigated the contribution of each factor of the phosphorus metabolism network by phosphorus diet intervention using Granger causality analysis^20^. Time-series clustering is one of the methods of cluster analysis utilizing time-series data, which is used in diverse scientific fields from gene expression data in biology to stock market analysis in finance^21^. In this study, we used hierarchical clustering, an approach to cluster analysis that builds a hierarchy of clusters, with the Ward method on eGFR data over time. The novelty of the present study lies in the combination of two different forms of machine learning. The time-series cluster analysis enabled us to identify an unexpected subgroup of eGFR trajectory from CKD patients such as Class 5 for whom the eGFR decline was relatively rapid compared to other classes, while LightGBM identified the important variables for predicting the results of cluster analysis.

LightGBM^22^ is a high-performance gradient boosting framework that offers faster training efficiency, lower memory requirements, and higher accuracy than extreme gradient boosting^23^ or other implementations. The LightGBM algorithm incorporates two novel techniques, gradient-based one-side sampling and exclusive feature bundling, enabling the algorithm to run faster while maintaining a high level of accuracy^22^. LightGBM allows missing values for prediction so imputation is not required, providing an advantage over conventional logistic regression modeling. We performed classification and regression analysis to elucidate relationship between each cluster of eGFR and the baseline characteristics of patients using SHAP, a method for interpreting the predictions of machine learning models^24^.

Recently, clinical investigations using machine learning models have dramatically increased, especially in the past 3 years. Some studies have used similar methods as this study in the field of nephrology. Quinn et al. analyzed patients with CKD using hierarchical clustering according to histological features and assessed the relationship between histopathology-based clusters and eGFR declines over time by a linear mixed model^25^. Dong et al. constructed a prediction model for the development of diabetic kidney disease within 3 years using machine learning models, including LightGBM, and identified predictors for diabetic kidney disease^26^. They compared the performance of seven machine learning algorithms and showed that the LightGBM model offered the highest AUC, sensitivity, positive predictive values, and negative predictive values. However, to the best of our knowledge, the present investigation is the first to apply both cluster analysis and a LightGBM algorithm to stratify patients according to the eGFR trajectory and predict 5-year transitions in eGFR among CKD patients with relatively preserved kidney function.

Figures 2 and 4 indicate that prediction of the eGFR trajectory by LightGBM mostly depends on baseline eGFR, with other features thought to be generally associated with CKD progression not having much impact compared with baseline eGFR under the care of nephrologists. The time course of eGFR in patients belonging to Classes 1–4 seemed to be parallel and showed less change in eGFR over 5 years compared with Class 5 (Fig. 2a). Interestingly, Classes 4 and 5 patients showed similar eGFRs at baseline, but Class 5 showed progression of kidney dysfunction while Class 4 maintained relatively preserved function during follow-up, although patients in both Classes 4 and 5 were under the care of nephrologists. To elucidate the difference between patients in Classes 4 and 5, we conducted additional analysis of the two classes using the same LightGBM algorithm and including a greater number of covariates). According to the results of the importance summary plot using Models 1 and 2 (Fig. 4a and Supplemental Fig. 3), we selected covariates as Model 3, which appeared to provide robust prediction, and actually the AUC of the ROC was highest for the Model 3 prediction (Fig. 5). As in Fig. 4a, baseline eGFR was the most important factor for prediction, and other baseline characteristics did not have much importance compared with baseline eGFR. Figure 7 indicates that patients with eGFR ≥ 45 mL/min/1.73 m^2^ but < 50 mL/min/1.73 m^2^ were at increased risk of being in Class 5, in which declines in eGFR were relatively rapid. Therefore, from the dataset of this cohort at least, we can say that preventing CKD progression would be difficult for patients with eGFR < 50 mL/min/1.73 m^2^ at baseline, regardless of comorbidities such as proteinuria or history of diabetes, which were generally identified as important factors in treating CKD. All participants included in the present study were under the care by the specialist of nephrology or diabetology and might be on the appropriate risk management such as blood pressure control. In fact, mean of baseline systolic blood pressure was 133.9 mmHg and 75.7% of the patients were treated ACE inhibitor or ARB in Class 5. However, the results of the present study indicated that baseline eGFR (cut-ff value of below 50 mL/min/1.73 m^2^) was the most important factor for prediction of rapid decline in eGFR (Class 5), therefore, patients with eGFR < 50 mL/min/1.73 m^2^ might be at high risk for CKD progression or ESKD despite intensive renal care by the specialist. We hypothesized that this eGFR threshold of 50 mL/min/1.73 m^2^ represented a so-called “point of no return”^27^, at least in this cohort of CKD patients. Our findings also suggest that physicians should refer patients to nephrologists and optimal reno-protective risk management is strongly required from early stage of CKD before eGFR reaches below 50 mL/min/1.73 m^2^ to prevent progression to ESKD.

Other than eGFR, RDW was the most important factor affecting the prediction of Class 4 or 5. Figure 7 indicates that patients with RDW ≥ 14.5% were at increased risk of being in Class 5. RDW is a measure of the heterogeneity of red blood cell volume^28^ and an elevated RDW is reportedly associated with ESKD in CKD patients via multiple factors^17,29^. An elevated RDW reflects a worsened status of patients, such as malnutrition, iron deficiency, or inflammation, and might have been important for the prediction via LightGBM in this study. However, close mechanism between elevated RDW and rapid decline in eGFR in these patients it is still unclear, therefore, it will be necessary to elucidate whether elevated RDW itself is directly related to increased risk of CKD progression or whether it is an indirect relationship reflecting malnutrition, or inflammation in the future studies.

Serum albumin, LDL-cholesterol, and hemoglobin were also recognized as affecting the results of prediction, although their importance was not as high as baseline eGFR or RDW. According to Fig. 7, patients with serum albumin < 3.5 g/dL, LDL-cholesterol < 100 mg/dL, and hemoglobin < 13.0 g/dL were at higher risk of being classified to Class 5. Serum albumin level reflects not only nutritional status or inflammation, but also the degree of proteinuria in CKD patients, which may have higher importance for prediction than a qualitative test for proteinuria alone. LDL-cholesterol level is affected by nutritional status and the use of antihyperlipidemic agents. The mechanism by which lower LDL-cholesterol increased the risk of being in Class 5 was unclear, since body mass index, rate of dyslipidemia and use of antihyperlipidemic medications did not differ significantly between Classes 4 and 5 (Table 2). Anemia has been reported as a risk factor for CKD progression^30,31^ and the present results support this contention.

Several limitations are apparent in this study. First, this study was conducted at a single institution, and the sample size was relatively small, so the results will not necessarily reflect the worldwide clinical environment. We must therefore analyze huge datasets from other countries and ethnicities to predict CKD progression more precisely in future studies. Second, we used only a single machine learning algorithm to predict the result of cluster analysis and did not compare results with those from other prediction models such as random forests, support vector machine or logistic regression. Although LightGBM is considered a superior performance algorithm, the interpretation of this study might have differed if other algorithms had been used. Validation studies are still needed to clarify the superiority of LightGBM compared to other algorithm method. Third, in the present study, patients were stratified into 5 classes with similar trajectories by cluster analysis based on 5-year changes in eGFR and the results suggested that reduction in eGFR was remarkedly larger in Class 5. It is also remarkedly crucial in clinical practice whether this class stratification is associated with mortality risk in these patients, although we could not assess these analyses in the present study. This point should be determined in future studies, as well. Forth, Patients without serum creatinine data were excluded, but those without urinary protein data were not excluded in the present study (n = 19). As conducting urinary tests were determined by their attending physician, the detailed reasons why the urinary tests were not conducting were unclear in these patients. Urinary protein has a significant effect on both diagnosis of CKD and its progression and missing data of urinary protein could affect the results, however, in LightGBM, missing values are ignored during a split and allocated to whichever side reduces the loss the most, so a little data missing dose not significantly affect the results.

Nevertheless, we have presented here a new approach using cluster analysis and a LightGBM algorithm to stratify patients according to the transition of eGFR and to identify important clinical features for the progression of CKD by prediction modeling. Identifying subgroups with rapid declines in eGFR such as Class 5 is difficult without cluster analysis, and LightGBM allows prediction with higher accuracy than conventional methods without imputation of lacking data. This approach should be applied with big data, including patients in other countries, to clarify the clinical time course of CKD and identify important clinical factors for preventing progression of CKD.

### Supplementary Information

Supplementary Legends.

Supplementary Figure 1.

Supplementary Figure 2.

Supplementary Figure 3.

Supplementary Tables.

## Data Availability

The data that support the findings of this study are available on request from the corresponding author. The data are not publicly available due to privacy or ethical restrictions.
