# Supplementary material for: Predicting CKD progression using time-series clustering and light gradient boosting machines
Source: Sci Rep. 2024 Jan 19;14:1723. doi: 10.1038/s41598-024-52251-9 (PMC10798962; doi:10.1038/s41598-024-52251-9)
Supplement: Supplementary file 1 — Supplementary Legends. [file 41598_2024_52251_MOESM1_ESM.docx]

**Supplemental Figure S1.** Importance matrix plot of the LightGBM model, representing the importance of each covariate (top 12 covariates in the model removing baseline eGFR from Model 1) for predicting classification of 5-year eGFR trajectory.

adjusted for age, sex, body mass index, smoking history, systolic blood pressure, diastolic blood pressure, histories of cardiovascular disease and diabetes, serum albumin, hemoglobin, qualitative test for proteinuria, and use of an ACE inhibitor or ARB.

GBM, gradient boosting machine, eGFR, estimated glomerular filtration rate; ACE, angiotensin-converting enzyme; ARB, angiotensin II receptor blocker.

**Supplemental Figure S2.** Importance matrix plot of the LightGBM model in Class 4 vs. Class 5 patients, representing the importance of each covariate (top 12 covariates in the model removing baseline eGFR from Model 3) for predicting classification of 5-year eGFR trajectory.

adjusted for age, sex, body mass index, smoking history, systolic blood pressure, diastolic blood pressure, history of cardiovascular disease, diabetes, LDL-cholesterol, serum albumin, hemoglobin, platelet count, MCHC, RDW, qualitative test for proteinuria, use of an ACE inhibitor or ARB, and use of a xanthine oxidase inhibitor.

GBM, gradient boosting machine, eGFR, estimated glomerular filtration rate; LDL, low-density lipoprotein; MCHC, mean corpuscular hemoglobin; RDW, red cell distribution width; ACE, angiotensin-converting enzyme; ARB, angiotensin II receptor blocker.

**Supplemental Figure S3.** Importance matrix plot of the LightGBM model, representing the importance of each covariate (top 14 covariates in Model 2) for predicting classification of 5-year eGFR trajectory.

Model 2 adjusted for age, sex, body mass index, obesity, smoking history, systolic blood pressure, diastolic blood pressure, pulse pressure, heart rate, history of cardiovascular disease, hypertension, diabetes, dyslipidemia, hyperuricemia, eGFR, corrected calcium, phosphorus, LDL-cholesterol, serum albumin, white blood cell count, hemoglobin, platelet count, MCV, MCH, MCHC, RDW, qualitative test for proteinuria, use of medicine (ACE inhibitor or ARB, calcium channel blocker, loop diuretic, thiazide diuretic, spironolactone, statin, xanthine oxidase inhibitor, aspirin, warfarin, or proton pump inhibitor).

LDL, low-density lipoprotein; MCV, mean corpuscular volume; MCH, mean corpuscular hemoglobin; MCHC, mean corpuscular hemoglobin concentration; RDW, red cell distribution width; ACE, angiotensin-converting enzyme; ARB, angiotensin II receptor blocker.
