# Supplementary figures and images for: Predicting CKD progression using time-series clustering and light gradient boosting machines
Source: Sci Rep. 2024 Jan 19;14:1723. doi: 10.1038/s41598-024-52251-9 (PMC10798962; doi:10.1038/s41598-024-52251-9)

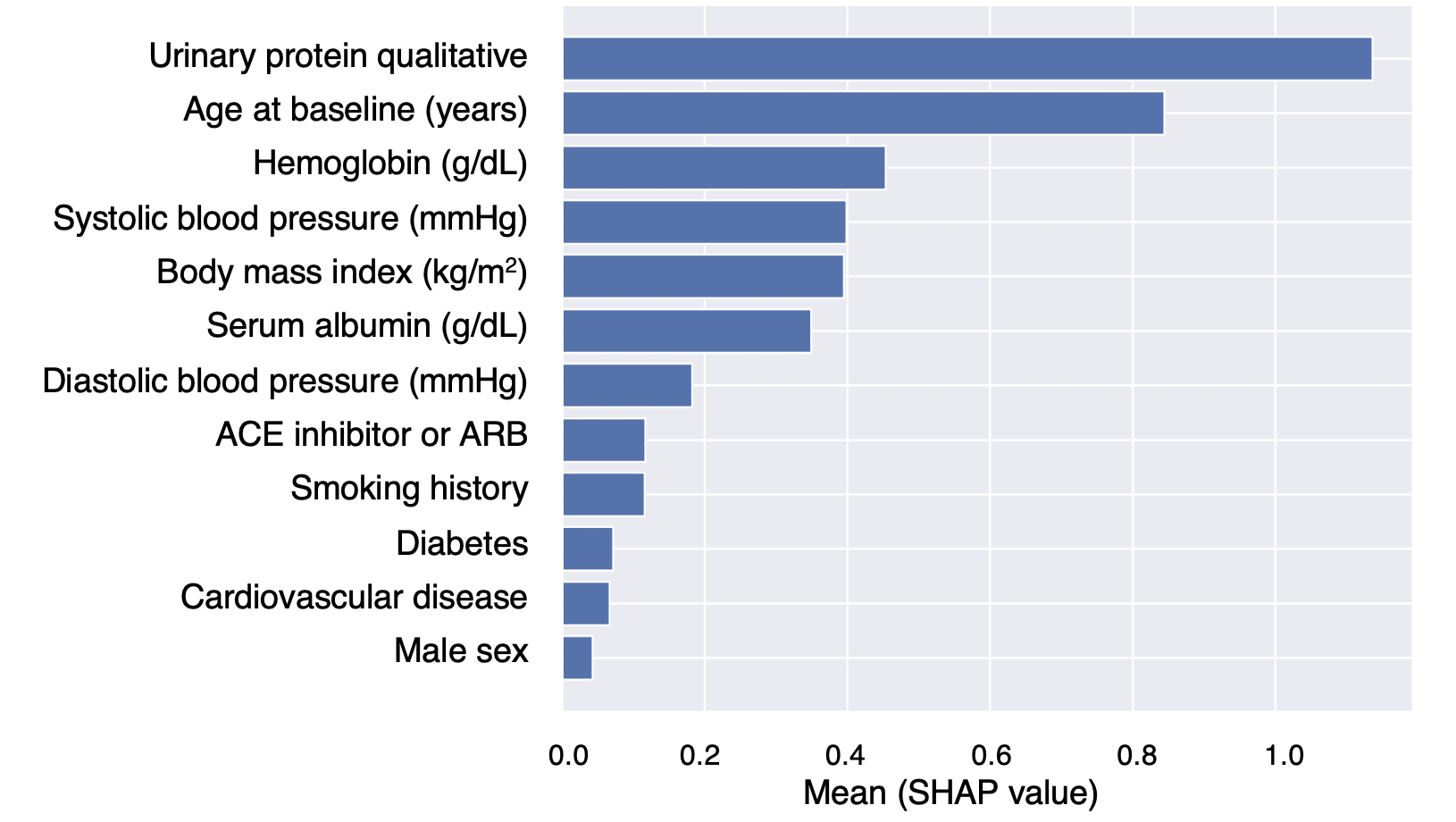

Supplement: Supplementary file 2 — Supplementary Figure 1. [file 41598_2024_52251_MOESM2_ESM.tif]

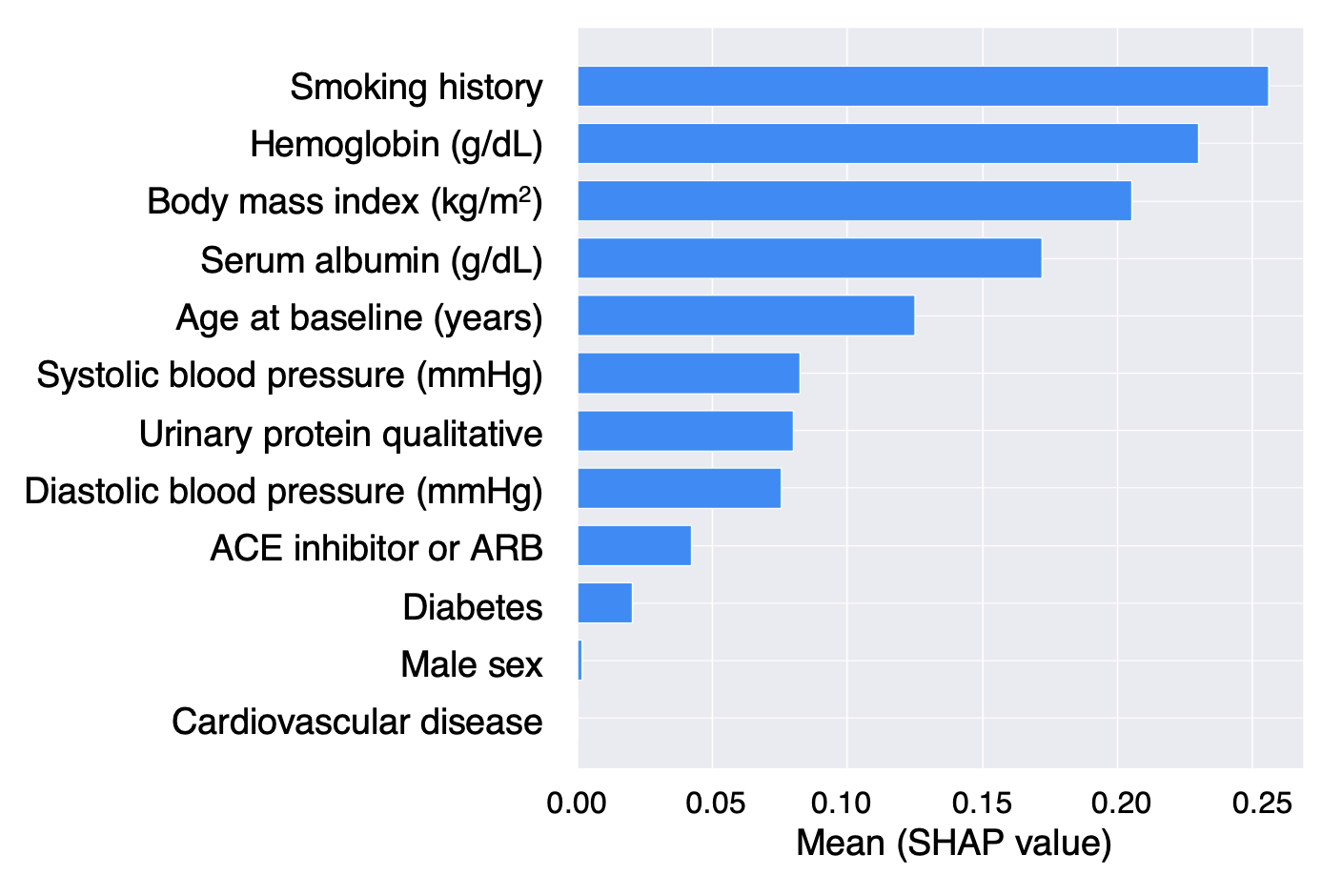

Supplement: Supplementary file 3 — Supplementary Figure 2. [file 41598_2024_52251_MOESM3_ESM.tif]

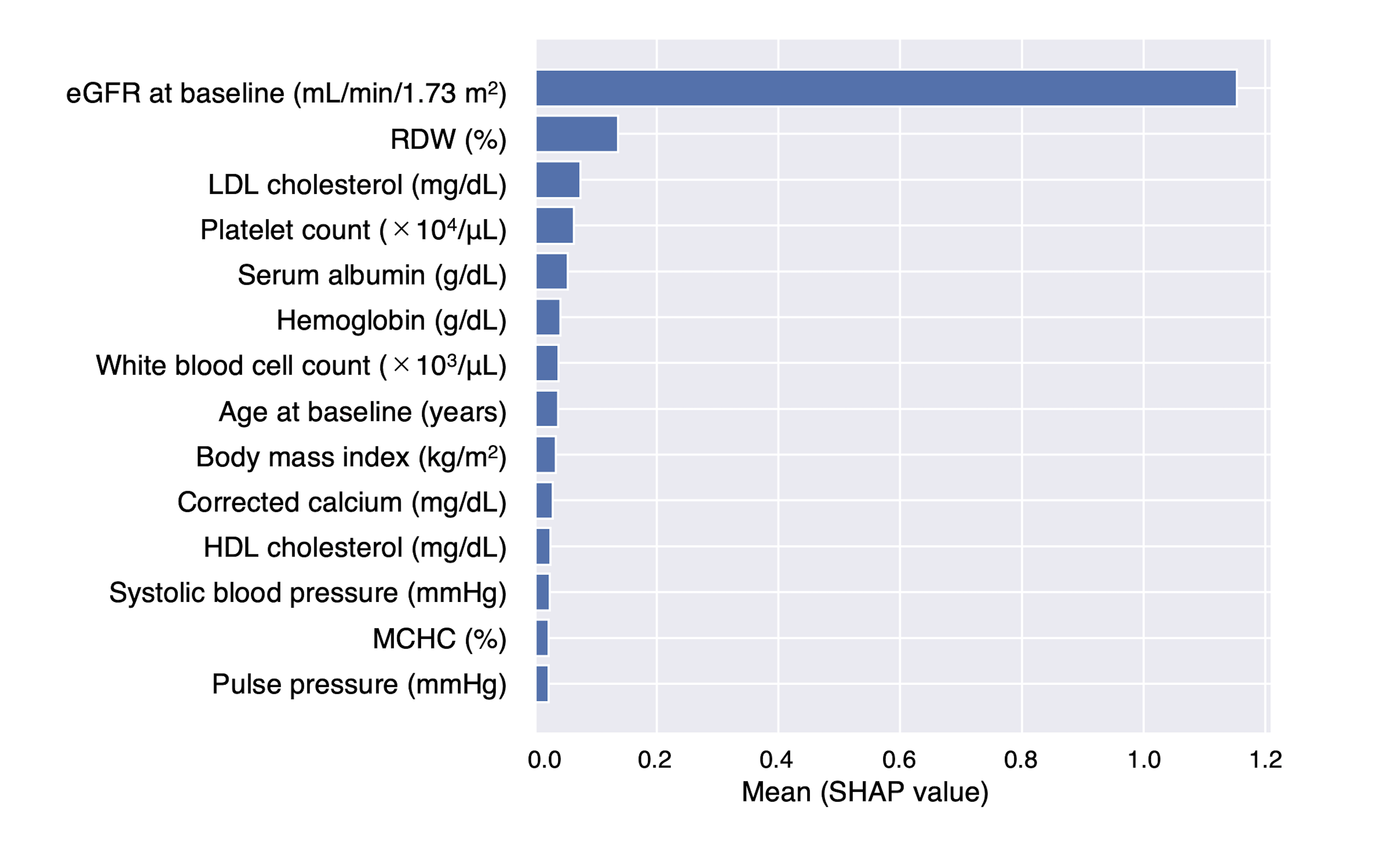

Supplement: Supplementary file 4 — Supplementary Figure 3. [file 41598_2024_52251_MOESM4_ESM.tif]
