# Supplementary material for: Predicting CKD progression using time-series clustering and light gradient boosting machines
Source: Sci Rep. 2024 Jan 19;14:1723. doi: 10.1038/s41598-024-52251-9 (PMC10798962; doi:10.1038/s41598-024-52251-9)
Supplement: Supplementary file 5 — Supplementary Tables. [file 41598_2024_52251_MOESM5_ESM.docx]

Table S1: Multi-class confusion matrix for Model 2

|  |  | Actual classification | | | | |  |
| --- | --- | --- | --- | --- | --- | --- | --- |
|  |  | Class 1 | Class 2 | Class 3 | Class 4 | Class 5 | Total |
| Predicted classification | Class 1 | 17 | 5 | 0 | 0 | 0 | 22 |
|  | Class 2 | 6 | 44 | 10 | 0 | 2 | 62 |
|  | Class 3 | 1 | 3 | 24 | 11 | 0 | 39 |
|  | Class 4 | 0 | 1 | 23 | 64 | 18 | 106 |
|  | Class 5 | 0 | 0 | 0 | 3 | 2 | 5 |
|  | Total | 24 | 53 | 57 | 78 | 22 | 234 |

Table S2: Multi-class confusion matrix for Model 3

|  |  | Actual classification | | | | |  |
| --- | --- | --- | --- | --- | --- | --- | --- |
|  |  | Class 1 | Class 2 | Class 3 | Class 4 | Class 5 | Total |
| Predicted classification | Class 1 | 18 | 5 | 0 | 0 | 0 | 23 |
|  | Class 2 | 5 | 45 | 7 | 0 | 2 | 59 |
|  | Class 3 | 1 | 3 | 23 | 12 | 0 | 39 |
|  | Class 4 | 0 | 0 | 26 | 63 | 19 | 108 |
|  | Class 5 | 0 | 0 | 1 | 3 | 1 | 5 |
|  | Total | 24 | 53 | 57 | 78 | 22 | 234 |
